# Influence of sealer and supplementary approach on filling material removal during endodontic retreatment

**DOI:** 10.1590/1807-3107bor-2024.vol38.0022

**Published:** 2024-07-15

**Authors:** Jáder Camilo PINTO, Fernanda Ferrari Esteves TORRES, Airton Oliveira SANTOS-JUNIOR, Karina Ines Medina Carita TAVARES, Juliane Maria GUERREIRO-TANOMARU, Mário TANOMARU-FILHO

**Affiliations:** (a) Universidade Estadual Paulista – Unesp, School of Dentistry, Department of Restorative Dentistry, Araraquara, SP, Brazil.

**Keywords:** Dental Pulp Cavity, Retreatment, Root Canal Preparation, Root Canal Filling Materials, X-Ray Microtomography

## Abstract

Both root canal sealer-based and supplementary protocols may influence removal of filling material during endodontic retreatment. Mesial root canals of extracted mandibular molars were prepared using HyFlex EDM 25/.08, and filled with a calcium silicate sealer (Bio-C Sealer), or an epoxy resin (AH Plus), using the single cone technique (n = 12). Retreatment was performed using ProDesign Logic (PDL) RT and PDL 35/.05. The specimens were randomly divided into two experimental groups (n = 12), and the sealers were distributed similarly. A supplementary protocol was performed with PDL 50/.01 or XP-endo Finisher. Root canal transportation and volume, in addition to the remaining filling material percentage were evaluated using high-resolution (5 µm voxel size) micro-CT. Statistical analysis was performed using t-tests (α = 0.05). Root canals filled with AH Plus presented high residual filling material (p < 0.05). Both protocols decreased residual volume of filling material in the apical third (p < 0.05). PDL 50/.01 increased the apical root canal volume (p < 0.05). No difference was observed between the systems regarding canal transportation (p > 0.05). In conclusion, AH Plus is more difficult to remove from the apical third than Bio-C Sealer. PDL 50/.01 and XP-endo Finisher enabled greater removal of filling materials in the apical third, in the retreatment of curved root canals, without promoting apical transport.

## Introduction

The removal of filling material in retreatment procedures is essential to the disinfection of the root canal system.^1^ Endodontic sealers based on calcium silicates are used due to their biocompatibility and induction of mineralization.^2^ However, the literature is controversial regarding the impact of calcium silicate–based sealers on endodontic retreatment.^3^ Filling material removal after retreatment of root canals filled with AH Plus (Dentsply DeTrey, Konstanz, Germany) or calcium silicate–based sealers was observed to be similar for both materials,^4-6^ However, removal of the filling material may be influenced by different sealers.^7^

Bio-C Sealer (Angelus, Londrina, Brazil) is a premixed, ready-to-use calcium silicate–based sealer. This sealer has shown cytocompatibility and mineralization,^8^ in addition to radiopacity and flow, in accordance with the ISO 6876 standard.^9^ Bio-C Sealer presents a low presence of voids, similar to AH Plus,^10^ thereby serving as an appropriate root canal filling.^11^ Removal of Bio-C Sealer combined with gutta-percha in retreatment has been evaluated in oval root canals.^12^ However, there are no studies investigating the retreatment of curved root canals filled with Bio-C Sealer.

The apical region of the root canal is a critical zone for continued infection of the root canal system.^13^ Apical enlargement can improve removal of the filling material.^14^ However, root canal preparation using nickel-titanium (NiTi) instruments with large diameter and taper in curved root canals can promote root canal transportation due to their low flexibility.^15^ ProDesign Logic 50/.01 (PDL - Easy Equipamentos Odontológicos, Belo Horizonte, Brazil) is a NiTi rotary instrument with Control Memory (CM) heat-treatment, large tip size and minimum taper, and was proposed to perform the apical enlargement in this study.^14^ This instrument significantly decreased the amount of filling material in the apical third of the curved lateral incisors.^14^Although it has been reported that NiTi files with CM heat-treatment enable apical enlargement in curved root canals,^16^ there are no studies evaluating apical transportation caused by CM instruments with tip size 50.

XP-endo Finisher (FKG Dentaire, La Chaux-de-Fonds, Switzerland) is a non-tapered rotary MaxWire NiTi instrument with tip size 25, which changes its shape according to the temperature.^17,18^This file effectively removes remaining filling material that accumulates.^19,20^ XP-endo Finisher and XP-endo Finisher R files are equally effective as an adjunct procedure adopted in endodontic retreatment.^20^ However, there is no investigation that compares filling material removal using XP-Endo Finisher versus apical enlargement, or that assesses apical transportation or volume increase after use of this finisher in curved root canals of molars.

The aim of the present study was to evaluate the influence of a ready-to-use calcium silicate–based root canal sealer compared with an epoxy resin–based sealer, on the removal of filling material during endodontic retreatment, and to compare filling material removal, apical transportation and volume increase of curved mesial root canals of mandibular molars filled with AH Plus versus Bio-C Sealer, after using size 50, .01 taper instrument or XP-endo Finisher. The first null hypothesis was that the obturation of the root canal with AH Plus or Bio-C Sealer would not impact the filling material removal. The second null hypothesis was that no differences would be observed in the amount of filling material, the transportation or the root canal volume using PDL 50/.01 or XP-endo files.

## Methodology

### Sample size calculation

G* Power 3.1.7 for Windows program (Heinrich-Heine-Universitat Dusseldorf, Dusseldorf, Germany) was used for the sample calculation. The t-test for two dependent groups was used with an alpha type error of 0.05 and beta power of 0.95 for all the variables: filing material removal^20^; root canal volume, 1.860;^16^ and root canal transportation, 3.111.^21^ A total of 12 specimens were indicated as being the ideal size required.

### Sample selection

All procedures were approved by the Dental School’s Ethics Committee (CEP no. 10411219.9.0000.5416). The study used human mandibular first and second molars previously stored in 0.1% thymol solution at 5°C. The inclusion criteria included two independent mesial root canals according to Vertucci’s Type IV classification,^22^ angle of curvature between 25° and 35°, in accordance with the Schneider method,^23^ and radius of curvature smaller than 10 mm, following the Pruett method.^24^ Teeth with complete apical formation, but without root fractures, calcifications or internal resorptions were also included. A digital system (RVG 6100; Kodak Dental Systems, NY) and a microtomography (micro-CT) device (SkyScan 1276; Bruker-microCT, Kontich, Belgium) were used to evaluate the parameter criteria. Micro-CT was applied at low resolution (35 µm voxel size) under the following settings: copper and aluminum filters, 87-millisecond exposure time, frame averaging of 3, 180° rotation around the vertical axis, rotational step of 0.5° at 80 kV and 300 µA. A total of twelve roots were selected, totaling twenty-four root canals, including the mesiobuccal and mesiolingual canals of each root.

### Root canal preparation

Conventional access cavities were performed, and the root canals were explored with a size #10 K-file (Dentsply Sirona Endodontics, Ballaigues, Switzerland). The working length (WL) was established at 1 mm short of the apical foramen. The preparation used HyFlex EDM (HEDM; Coltene/Whaledent, Altstätten, Switzerland) 10/.05, operated by the VDW SILVER electric motor (VDW, Munich, Germany) in rotary motion at 300 rpm and 1.8 Ncm torque, with in-and-out movements up to the WL. Then, HEDM 25/.08 was used at 500 rpm and 2.5 Ncm torque, as described above. Each root canal was irrigated with 5 mL of 2.5% sodium hypochlorite (NaOCl). Passive ultrasonic irrigation (PUI) was performed in all the root canals with the Irrisonic ultrasonic tip (Helse Ultrasonic, Santa Rosa de Viterbo, Brazil), activated with an Ultrawave XS ultrasonic device (Ultradent, South Jordan, USA), using a power of 10% and frequency of 50 Hz, according to the manufacturer’s recommendation. The ultrasonic tips were positioned 2 mm short of the WL and activated for 20 seconds, alternating 2.5% NaOCl and 17% EDTA. A 2 mL aliquot of irrigation solution was used in each cycle. The final irrigation used 5 mL of distilled water.

### Root canal filling

A stratified, random sampling statistical method was used to divide the root canals into two experimental groups, considering the post-preparation volume (n = 12). The root canals were dried with paper points, and filled with gutta-percha cones 25/.08 (Coltene/Whaledent, Langenau, Germany) and an epoxy resin–based sealer (AH Plus; Dentsply DeTrey, Konstanz, Germany), or a calcium silicate–based sealer (Bio-C Sealer; Angelus, Londrina, Brazil), using the single cone technique. AH Plus was inserted into the root canal with a Lentulo spiral #25 (Dentsply Maillefer) coupled to a low speed motor (Micromotor N270 and Counter-angle, Dabi-Atlante, Ribeirão Preto, SP, Brazil). Bio-C Sealer was injected into the root canal at 4 mm short of the WL, using the syringe and plastic needles provided by its manufacturer. The access cavities were filled with Coltosol (Vigodent, Rio de Janeiro, Brazil). The samples were stored at 37ºC and 95% relative humidity for 1 week to allow the sealers to set completely.

### Root canal retreatment

Retreatment used the VDW SILVER electric motor in rotary motion at 600 rpm and 3 Ncm torque. The files were used with in-and-out movements up to the WL. ProDesign Logic RT (PDLRT - Easy Equipamentos Odontológicos, Belo Horizonte, Brazil) 30/10 was used to remove the filling in the cervical third; PDLRT 25/.08 was used in the middle third; and PDLRT 20/.06 was used in the apical third. The root canals were enlarged with PDL 35/.05, as described above. Each root canal was irrigated with 8 mL of 2.5% NaOCl (2 mL for each instrument). Final irrigation was performed with 2.5 mL EDTA under agitation for 3 minutes, followed by 5 mL of distilled water.

### Supplementary retreatment approach

The mesial root canals of the specimens were randomly divided into two experimental groups (n = 12) by stratified random sampling, according to the remaining root canal filling volume after retreatment, and considering equal distribution of the sealers (AH Plus and Bio-C Sealer) between the groups.

*PDL 50/.01*: the instrument was operated using the VDW SILVER electric motor in rotary motion, at a speed of 350 rpm and 1 Ncm torque, with in-and-out movements up to the WL. The root canals were irrigated with 2 mL of 2.5% NaOCl. Final irrigation was performed as described previously.

XP-endo Finisher: the instrument was operated using the VDW SILVER electric motor in rotary motion, at a speed of 800 rpm and 1 Ncm torque. Each canal was filled with 1 mL of 2.5% NaOCl, and the XP-endo file was inserted without rotation. Then, rotation was initialized (800 rpm and 1 Ncm), and the instrument was activated for 30 seconds using slow and gentle 7-8 mm lengthwise movements up to the WL. This procedure was repeated twice. Final irrigation was performed as described previously.

### Micro-CT analysis

The specimens were scanned at high resolution (5 µm voxel size) in a micro-CT device (SkyScan 1272. Bruker, Kontich, Belgium), before and after retreatment procedures, under the following settings: copper filter, 180° rotation around the vertical axis, and rotational step of 0.2° at 100 kV and 100 µA. The images were reconstructed using NRecon software (V1.6.10.4; Bruker, Belgium), and superimposed with geometric alignment using DataViewer software (V.1.5.1, Bruker, Belgium). The quantitative analysis was performed using CTAn software (V.1.14.4, Bruker, Belgium). Representative images were performed with models obtained by CTVox software (v.3.2; Bruker-microCT).

Root canal volume and remaining filling material were quantified after performing the retreatment steps. The gray scale range needed to recognize each object under study was determined in a density histogram by using the global threshold method. The following formula was used to obtain the percentage of remaining filling material: [Percentage of remaining filling material = (filling material after retreatment x 100 / filling material before retreatment)]. The analyses were performed in the apical third of the root canals, considering 4 mm.

**Figure 1:**
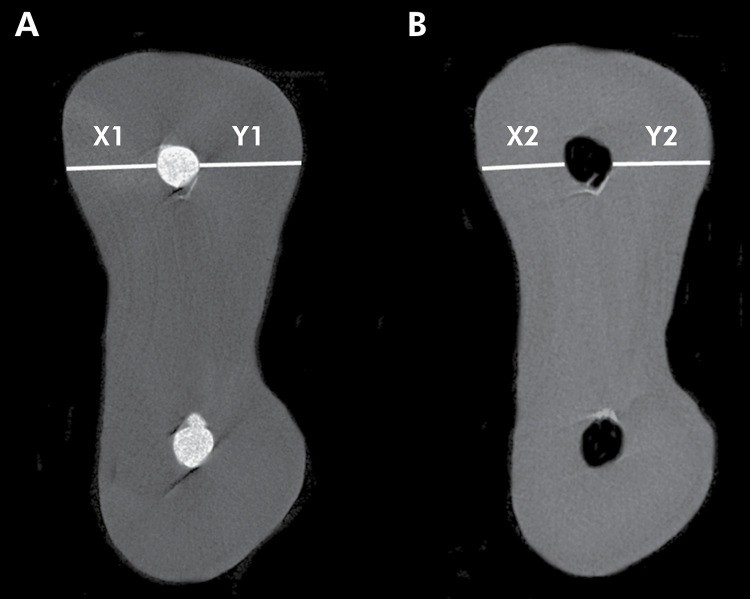
Representative cross-sectional micro-CT images showing how apical transportation was calculated: (A) before and (B) after root canal retreatment.

The apical transportation was measured in millimeters using transverse cross-section images of the roots, obtained before and after retreatment procedures (Figure 1). Ten cross sections were measured in the apical third of each root canal, determined by the arithmetical mean value. The root canal transportation was obtained using the following equation: (X1−X2)−(Y1−Y2) .^25^ X1 represents the shortest distance between the mesial edge of the root and the canal before instrumentation; Y1 represents the shortest distance from the inside of the curved root to the periphery of the canal, before instrumentation; X2 represents the shortest distance from the outside of the curved root to the periphery of the instrumented canal; and Y2 represents the shortest distance from the inside of the curved root to the periphery of the instrumented canal.

### Statistical analysis

The normality of the data was tested using the Shapiro-Wilk test. The paired t-test was used for comparison of root canals before and after performing the supplementary cleaning approach. The non-paired t-test was used for comparison of the groups. The level of significance was set at 5% for the entire analysis.

## Results

**Figure 2:**
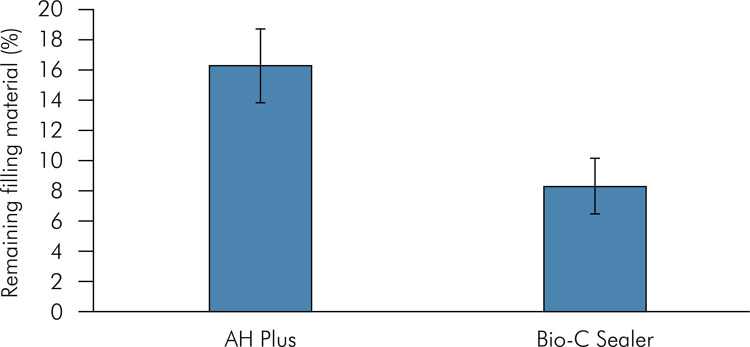
Means and standard deviations of residual filling material (%) in the apical third of the root canals filled with AH Plus or Bio-C Sealer. There is a significant intragroup difference (p < 0.05).

**Figure 3:**
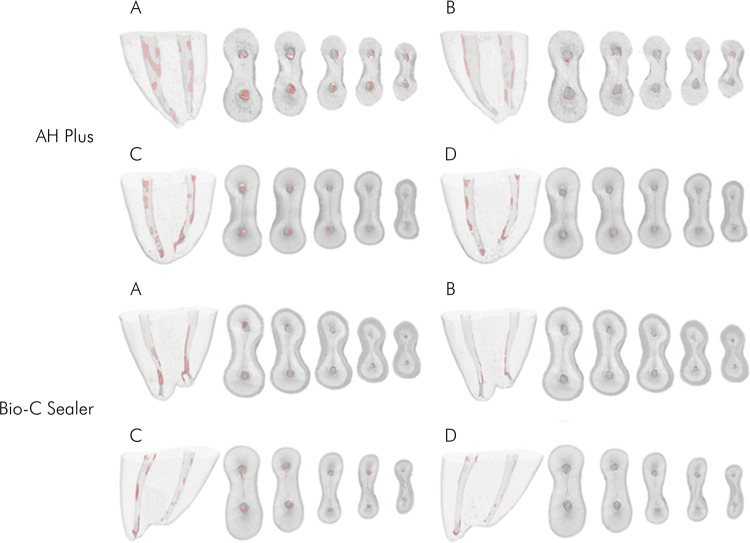
Representative 3D reconstructions of images obtained by micro-CT showing (in pink) the remaining filling material in the apical third of mesial canals of mandibular molars (A) before PDL (B) after PDL (C) before XP-endo Finisher, and (D) after XP-endo Finisher.

The root canals filled with AH Plus showed a mean and standard deviation of 16.32 ± 3.44 regarding the percentage of remaining filling material, whereas the canals filled with Bio-C Sealer showed 8.33 ± 2.44 (p < 0.05) (Figure 2). The volume of the remaining filling material was similar between the groups, before and after performing the supplementary cleaning approach with PDL 50/.01 or XP-endo (p > 0.05). Both files decreased the volume of remaining filling material in the apical third (p < 0.05) (Table 1, Figure 3). PDL 50/.01 increased the root canal volume in the apical third (p < 0.05) (Table 2). Both supplementary approaches maintained the root canal trajectory, with no difference between them (p > 0.05) (Table 3).

**Table 1:** Residual filling material (%) in the apical third of the root canals before and after the use of ProDesign Logic 50/.01 or XP-endo Finisher files and the reduction of residual filling material (%) (mean and standard deviation).

Variable	Before additional cleaning method	After additional cleaning method	Reduction in residual filling material
PDL 50.01	13.26 ± 3.36^a^	7.50 ± 2.13^b^	48.55 ± 6.70
XP-endo Finisher	10.61 ± 2.33^a^	6.33 ± 1.64^b^	53.50 ± 9.64

Different lowercase letters on same line represent significant differences intragroup (p < 0.05). There was no significant difference intergroup (p > 0.05).

**Table 2:** Root canal volume (mm3) in the apical third of the root canals before and after the use of ProDesign Logic 50/.01 or XP-endo Finisher files, and root canal volume increase (%) (mean and standard deviation).

Variable	Before additional cleaning method	After additional cleaning method	Reduction in residual filling material
PDL 50.01	0.81 ± 0.24^aA^	1.04 ± 0.32^aB^	29.36 ± 8.87a
XP-endo Finisher	0.80 ± 0.24^aA^	0.83 ± 0.25^bB^	3.89 ± 1.93^b^

Different lowercase letters on same column represent significant differences intergroup (p < 0.05). Different capital letters on same line represent significant differences intragroup (p < 0.05).

**Table 3:** Transportation (mm) in the apical third of the root canals before and after the use of ProDesign Logic 50/.01 or XP-endo Finisher files (mean and standard deviation).

Variable	Before additional cleaning method	After additional cleaning method
PDL 50.01	0.038 ± 0.015	0.057 ± 0.035
XP-endo Finisher	0.039 ± 0.020	0.053 ± 0.021

There were no significant differences intragroup or intergroup (p > 0.05).

## Discussion

The first null hypothesis was rejected, because the root canals obturated with Bio-C sealer showed less remaining filling material, compared with AH Plus. The removal of the calcium silicate–based sealers was greater compared with that of AH Plus in a previous investigation.^4^ The physicochemical properties of calcium silicate–based sealers may influence their removal during retreatment procedures.^7^ Bio-C Sealer has higher solubility than AH Plus,^10^ a feature which could contribute to its removal by the mechanical action of the files, and the physical effect of irrigation solutions. Conversely, the high bond strength of AH Plus could promote its adhesion to the dentine walls,^26,27^ thus making it difficult to remove.

Our study also compared the efficacy of two approaches for additional retreatments. To this end, the root canals filled with AH Plus and Bio-C sealer were randomly divided into two experimental groups, seeking equal distribution of the sealers between the groups, in order to maintain the proper sample size after retreatment. In so doing, the influence of the sealer could no longer be considered. Thus, a limitation of the present study stems from the inability to define the influence of different sealers in the supplementary retreatment approach. The second null hypothesis was partially rejected, because both PDL 50/.01 and XP-endo Finisher improved the removal of remaining filling material in the apical third of curved mesial root canals of mandibular molars equally well. Our findings corroborate those of previous studies that have shown XP-Endo Finisher files as improving removal of filling materials during retreatment.^5,20,28^ The MaxWire alloy used to produce the XP-endo Finisher files allows the files to expand at body temperature.^29^ In so doing, the instrument tip is forced against the root canal wall, causing the elliptical part of the file to be compressed by the resistance imposed by canal anatomy.^30^ The mechanical action of the tip in the root walls can dislodge the root filling material, which is then removed during canal irrigation.

Our findings showed that PDL 50/.01 promoted a higher increase in root canal volume in the apical third than XP-endo Finisher. Large apical preparations increase the ability of the instrument to touch root canal surfaces,^31^ thus improving the removal of remaining filling material. In addition, apical enlargement improves the physical effect of the irrigating solution,^32^thus allowing better apical cleaning,^33^ and enhancing root canal disinfection significantly.^34^ However, there is a concern regarding preparations using files with a larger tip and taper, as regards the risk of excessive removal of dentin, leading to lower root strength.^35^ The root canal preparation for retreatment using a size 35/.05 taper provides a 0.55 mm diameter at 4 mm from the apical preparation. Therefore, it can be argued that the PDL 50/.01 file did not promote dentinal removal in the cervical and middle thirds, since this file has a 0.54 mm diameter at 4 mm from the apical preparation.

Root canal transportation, perforations or excessive wear of root canal walls can negatively affect the outcome of endodontic treatments.^36^ In the current study, retreatment with a 35/.05 apical size taper, and the supplementary approach using the PDL 50/.01 or XP-endo Finisher, did not result in canal transportation in the critical apical area. The mean transportation observed in this study ranged from 0.038 to 0.057 mm, which is below the acceptable limit of 0.15 mm.^37^ Heat-treated NiTi instruments are more flexible, and do not promote significant root canal transportation,^16,38^ even in retreatment procedures.^39^ The CM-wire alloy of the instruments used in this study may have contributed to the retreatment of curved root canals, owing to its fatigue resistance and flexibility. Even though the PDL 50/.01 has a large tip, its small taper makes the instrument more flexible.^40^ The XP-Endo Finisher has a small core tip size and zero taper, resulting in high flexibility, and ability to clean the dentine without changing the original root canal morphology.^17^

The variety of different endodontic calcium-silicate sealers introduced on the market stresses the importance of evaluating their impact on retreatment procedures. The results of the present study demonstrated the enhanced removal of filling materials with the PDL 50/.01 and XP-Endo Finisher files. Bio-C Sealer showed greater retractility. In addition, the research showed that both systems that were evaluated (XP-Endo Finisher and PDL 50/.01) performed similarly in removing filling material from the apical third, and in preventing root canal transportation. Although the taper of PDL 50/.01 makes it effective only in the apical root canal third, the apical increase that it promotes can improve root canal disinfection. Both supplementary approaches tested can be indicated for cleaning the apical third of curved root canals.

## Conclusions

AH Plus is more difficult to remove from the apical third than Bio-C Sealer. PDL 50/.01 and XP-endo Finisher provided better removal of filling materials in the apical third, in the retreatment of curved root canals, without promoting apical transport.
